# Effect of traditional Chinese exercises on motor function, balance, gait, and quality of life in patients with Parkinson’s disease: a systematic review and meta-analysis

**DOI:** 10.3389/fneur.2025.1708466

**Published:** 2026-01-14

**Authors:** Song Yapei, Li Liang, Fan Tonggang, Huang Xiaoyan, Zhao Chun'ai

**Affiliations:** 1 Shanghai Institute of Tourism, Shanghai, China; 2 School of Wushu, Shanghai University of Sport, Shanghai, China; 3 Shanghai Research Institute of Sports Science, Shanghai Anti-Doping Agency, Shanghai, China

**Keywords:** traditional Chinese exercises, Parkinson’s disease, motor function, balance, gait, quality of life, meta-analysis

## Abstract

**Introduction:**

Parkinson’s disease (PD) is a progressive neurodegenerative disorder characterised by progressive impairments in motor function, balance, and gait, which significantly compromise activities of daily living and overall quality of life. Traditional Chinese exercises (TCEs), recognised as a mind–body, non-pharmacological therapy, are frequently incorporated into the clinical management of PD. Despite their widespread use, the optimal therapeutic effects and ideal exercise prescriptions for TCEs remain uncertain. Therefore, we aimed to comprehensively evaluate the impact of TCEs on motor function, balance, gait, and quality of life in patients with PD through a systematic review and meta-analysis, with the goal of providing evidence-based guidance for the selection of the most suitable exercise regimen.

**Methods:**

This study adhered to the Preferred Reporting Items for Systematic Reviews and Meta-Analyses (PRISMA) guidelines, utilising the PICOS framework to systematically search seven databases—CNKI, Wanfang, VIP, PubMed, Embase, Cochrane Library, and Web of Science—for randomised controlled trials (RCTs) published from their inception up to October 2025. Two independent authors screened all eligible studies, and study quality and risk of bias were assessed using the criteria outlined in the Cochrane Handbook version 5.1. Extracted data were statistically analysed using RevMan 5.4 software, calculating the mean difference (MD) or standardised mean difference (SMD) with 95% confidence intervals (CIs) and evaluating heterogeneity (I^2^) among the studies.

**Results:**

Thirteen RCTs involving 859 patients were ultimately included in the analysis1. The meta-analysis results showed that, compared with the usual control group, TCEs significantly improved patients’ motor function (Unified Parkinson’s Disease Rating Scale part III, UPDRS-III, MD: −4.30, 95% CI: −5.90 to −2.70, *p* < 0.00001, *I*^2^ = 71%) and balance function (Berg Balance Scale, MD: 2.63, 95% CI: 1.30–3.96, *p* = 0.0001, *I*^2^ = 0%; Timed Up and Go Test, TUGT, MD: −1.19, 95% CI: −2.03 to −0.35, *p* = 0.005, *I*^2^ = 65). However, the improvements observed in gait function (stride length, *p* = 0.07; cadence, *p* = 0.85; and gait velocity, *p* = 0.77) and quality of life (PDQ-39, *p* = 0.11) were not statistically significant. Necessary subgroup analyses were conducted, with results indicating the following: for motor function (UPDRS-III), the optimal exercise type was Qigong (MD = −4.66), with an optimal exercise duration of ≤12 weeks (MD = −4.25) and frequency of >3 times per week (MD = −4.66). Furthermore, the independent practise of TCEs proved to be the most effective intervention method (MD = −5.66). For balance function (TUGT), the optimal exercise type was Qigong (MD = −2.47), with an optimal exercise duration of ≤12 weeks (MD = −2.2) and frequency of > 3 times per week (MD = −2.47).

**Conclusion:**

TCEs can serve as an effective adjunct to conventional therapy, significantly improving motor and balance functions in patients with PD. Based on these findings, we recommend Qigong as the preferred modality for early- and moderate-stage PD patients, with an optimal initial prescription involving a duration of ≤12 weeks, a frequency of > 3 times per week, and the independent practise of TCEs. However, studies on the effects of TCEs on gait function and quality of life are limited.

## 1 Introduction

Parkinson’s disease (PD) is the second most common neurodegenerative disorder and the most rapidly growing neurological disease worldwide (1). It is a systemic neurodegenerative condition that affects interconnected neural networks, resulting in a distinct set of motor and non-motor symptoms, including resting tremor, bradykinesia, muscle rigidity, and balance impairment, which severely affect day-to-day activities and health-related quality of life (2). The World Health Organization (WHO) estimates that by 2040, neurodegenerative diseases, including PD, will surpass cancer-related deaths and become the second leading cause of mortality globally (3). Currently, levodopa is considered the primary and preferred medication for the clinical management of PD (4); however, its long-term use is frequently associated with reduced efficacy, “on–off” phenomena, and motor complications, imposing a substantial psychological and financial burden on patients and their families, thereby reducing their quality of life (5). Therefore, the identification of alternative and adjunct therapies is crucial.

Recent studies have indicated that multimodal non-pharmacological interventions, especially physical activity, are highly effective in managing symptoms such as motor impairment, balance dysfunction, and gait abnormalities in patients with PD (6). Traditional Chinese exercises (TCEs) such as Tai Chi and Qigong are rooted in the holistic concept of life in Traditional Chinese Medicine (TCM), integrating wellness and health preservation to strengthen the body and prevent diseases (7). TCEs are commonly employed in the clinical management of PD as a mind–body non-pharmacological therapy, and are increasingly favoured by patients because of their gentle and slow nature, low cost, and lack of side effects.

Although multiple studies have confirmed that TCEs, particularly Tai Chi and Qigong, can substantially improve motor skills, walking function, and balance (8, 9), their specific efficacy remains a subject of debate. For instance, Mustafaoglu et al. noted that qigong was the most effective in improving walking speed, but its effects on functional activities, balance, and quality of life were insignificant (10). Furthermore, the effectiveness of different intervention strategies is inconsistent; for example, Tai Chi combined with medication did not show better improvement in gait or quality of life than medication alone (11). More importantly, existing systematic reviews lack in-depth subgroup analyses addressing critical parameters such as exercise types, duration, frequency, and intervention methods. Therefore, the most advanced clinical exercise intervention guidelines for patients with Parkinson’s disease remain unclear. Given this research gap, the present study aimed to systematically review and meta-analyse randomised controlled trials published until October 2025 to comprehensively evaluate the latest effects of TCEs on motor function, balance, gait function, and quality of life in patients with PD, providing an in-depth discussion and reliable evidence for developing suitable clinical exercise intervention programmes for this population.

## 2 Materials and methods

### 2.1 Search strategy

This study adhered to the Preferred Reporting Items for Systematic Reviews and Meta-Analyses (PRISMA) guidelines, applied strict inclusion and exclusion criteria, and was registered on the PROSPERO platform (ID: CRD420251137494). We searched seven databases, CNKI, Wanfang, VIP, PubMed, Embase, Cochrane Library, and Web of Science, from their inception until October 2025. The following keywords were used: “Parkinson’s Disease” or “Parkinsonism” or “Parkinson”; and “Tai-ji” or “Tai Chi” or “Taiji” or “Taijiquan” or “Qigong” or “Ch’i Kung” or “Qi Gong” or “Yijinjing” or “Wuqinxi” or “Liuzijue” or “Baduanjin” or “Traditional Chinese Medicine Exercise” or “TCM Exercise.” These two sets of keywords were combined using Boolean operators (“AND” and “OR”), such as: (Parkinson’s Disease OR Parkinson) AND (Tai Chi OR Qigong). Literature published in English or Chinese were also included in the analysis.

### 2.2 Inclusion and exclusion criteria

The inclusion and exclusion criteria for this study were developed using the PICOS (Population, Intervention, Comparator, Outcome, and Study Design) framework, which outlines both inclusion and exclusion criteria (Table 1).

**Table 1:** PICOS inclusion and exclusion criteria.

Category	Inclusion criteria	Exclusion criteria
Population	Adults (>18 years old) diagnosed with PD classified as Hoehn–Yahr stages 1–4, or meeting the Chinese PD Diagnostic Criteria (2016 edition) or the UK Brain Bank PD Diagnostic Criteria. No limitations were applied to sex and disease duration	Patients with other severe movement-limiting diseases besides PD (e.g., hypertension, heart disease, severe osteoarthritis, etc.); Healthy elderly individuals
Intervention	The intervention group received any form of standalone TCE (e.g., Tai Chi, Qigong) or TCE combined with stable medication (Jieyu Qingxin Tang) or rehabilitation robots	Did not focus on Traditional Chinese exercises
Comparator	The control group received usual care, no intervention, or no regular exercise	No control group, or the control group received other exercise interventions
Outcomes	The primary outcome was UPDRS-III. Secondary outcomes included the Berg Balance Scale, TUGT, PDQ-39, stride length, cadence, and gait velocity	Lacked the UPDRS-III outcome measure or had incomplete data
Study design	Randomised controlled trials published in Chinese or English	Systematic reviews, general reviews, conference papers, and other non-randomised controlled trials (non-RCTs)

### 2.3 Data extraction

Literature screening and data extraction were independently performed by two researchers (SYP and LL). First, duplicate records from different databases were merged using EndNote X9 software. Preliminary screening was conducted based on the titles and abstracts to exclude studies that did not meet the inclusion criteria. For eligible articles, full texts were downloaded for a second screening. In cases of disagreement regarding study inclusion or exclusion, a third author (FTG) made the final decision after discussion to reach consensus.

Data were extracted using a predesigned standardised form that included the first author’s name, publication year, country, sample size, patient age, intervention, comparator, outcome measures, and intervention duration.

### 2.4 Quality and risk of bias assessment

Two researchers (LL and HXY) independently assessed the quality of the included studies using the Cochrane Risk of Bias tool, which discusses seven domains of bias based on the Cochrane Handbook recommendations. The seven dimensions used to evaluate literature quality included random sequence generation, allocation concealment, blinding, and data integrity as follows: (1) random sequence generation, (2) allocation concealment, (3) blinding of participants and personnel, (4) blinding of outcome assessment, (5) incomplete outcome data, (6) selective reporting, and (7) other biases. Each risk of bias category was rated as “low risk,” “high risk,” or “unclear.” The two authors agree with the Cochrane bias assessment.

### 2.5 Data analysis

Data integration and analysis were conducted using Review Manager (RevMan) 5.4 software. For continuous variables, mean difference (MD) or standardised mean difference (SMD) was used as the effect size with a 95% confidence interval (CI). Heterogeneity was assessed using the I^2^ statistic, with values of 0–25% indicating low heterogeneity, 25–50% indicating moderate heterogeneity, and >50% indicating high heterogeneity. A fixed-effects model was used when I^2^ was <50% and a random-effects model was used when I^2^ was >50%. For analyses with high heterogeneity, sensitivity analysis was performed by sequentially excluding studies to explore the possible sources of heterogeneity. When necessary, research variables, such as exercise type, duration, and frequency and experimental group type, were used as grouping variables for the subgroup analyses to identify the sources of heterogeneity. Finally, funnel plots were generated to assess publication bias and verify the robustness of the results. Asymmetry in the plot suggests potential publication bias. Statistical significance was set at *p* < 0.05.

## 3 Results

### 3.1 Characteristics of included studies

**Figure 1:**
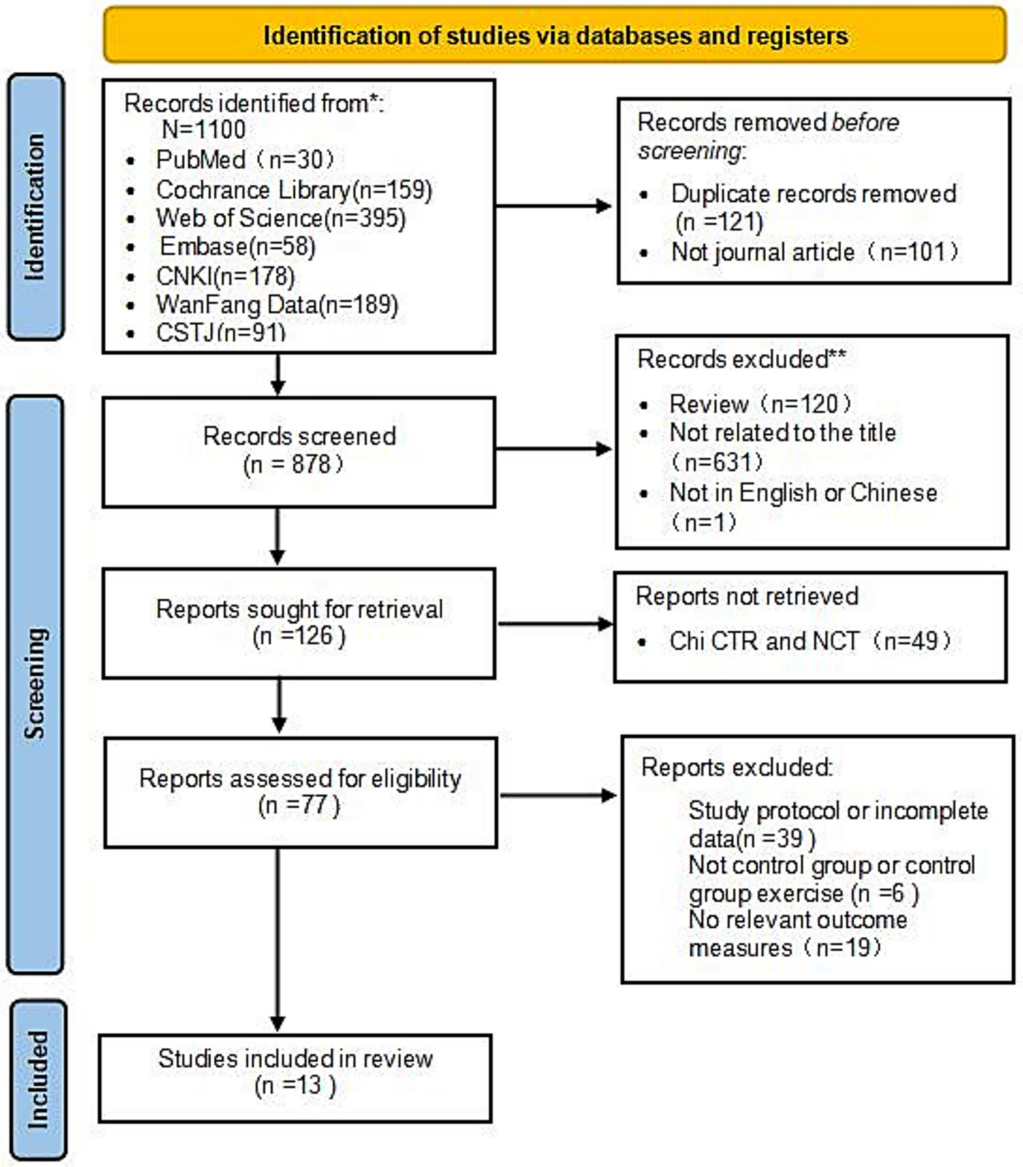
Flowchart of literature search.

A total of 1,100 articles were initially retrieved from the seven databases. After a stepwise screening process, 13 randomised controlled trials (RCTs) involving 859 patients with PD were included in the analysis. The search process is illustrated in Figure 1.

Thirteen articles [Jiang et al. (12); Lu et al. (13); Liu et al. (14); Liu et al. (15); Chen et al. (16); Li et al. (17); Wang et al. (18); Gao et al. (19); Xiao et al. (20); Li et al. (21); Vergara-Diaz et al. (22); Li et al. (23); Yin et al. (24)] investigated the effects of TCEs interventions on motor function, balance function, gait function, and quality of life in patients with PD. All the studies were published between 2014 and August 2025, with seven articles in Chinese and six in English. The intervention types included Tai Chi (4 studies) and Qigong (8 studies). The control group primarily received usual care or no exercise intervention. Specific information is presented in Table 2.

**Table 2:** Characteristics of included studies.

Author (year)	Country	T/C	Age		Intervention	Control	Outcomes	Duration
			CT					
Jiang and Bi (2023) (12)	China	20/20	59.45 ± 5.45	60.60 ± 6.75	Qigong	Usual care	①④	8 weeks, 5 times/week, 30 min/session
Lu et al. (2024) (13)	China	30/30	66.57 ± 6.82	66.70 ± 6.73	Qigong	Usual treatment	①	4 weeks, 5 times/week, 30 min/session
Liu et al. (2019) (14)	China	104/102	60.15 ± 9.75	61.43 ± 10.12	Qigong	Usual care	①③	48 weeks, 5 times/week, 30 min/session
Liu et al. (2017) (15)	China	23/18	57.1 ± 5.7	57.2 ± 8.0	Qigong	Usual care	①②	10 weeks, 5 times/week, 60 min/session
Chen et al. (2024) (16)	China	30/30	55.25 ± 6.85	56.10 ± 7.76	Qigong	Usual treatment	①③④	4 weeks, 5 times/week, not reported
Li et al. (2017) (17)	China	42/38	67.78 ± 5.36	65.25 ± 6.37	Tai Chi	Usual treatment	①⑤⑦	16 weeks, 3 times/week, 60 min/session
Wang et al. (2023A) (18)	China	15/15	67.13 ± 8.33	72.07 ± 8.33	Tai Chi	No intervention	①②	12 weeks, 3 times/week, 40 min/session
Wang et al. (2023B) (18)	China	15/15	67.13 ± 8.33	69.80 ± 6.90	Tai Chi	No intervention	①②	24 weeks, 3 times/week, 60 min/session
Gao et al. (2014) (19)	China	37/39	68.28 ± 8.53	69.54 ± 7.32	Tai Chi	No intervention	①②③	12 weeks, 3 times/week, 60 min/session
Xiao et al. (2015) (20)	China	45/44	66.52 ± 2.13	68.17 ± 2.27	Qigong	Usual activity	①②③⑤⑦	24 weeks, 4 times/week, 45 min/session
Li et al. (2024) (21)	China	27/27	60.4 ± 11.52	65.59 ± 9.16	Qigong	Usual activity	①④	4 weeks, 5 times/week, 40 min/session
Vergara-Diaz et al. (2018) (22)	United States	12/14	62 ± 7.77	65.7 ± 3.86	Tai Chi	Usual care	①②④	24 weeks, 2 times/week, 30 min/session
Li et al. (2022) (23)	China	15/16	63.2 ± 6.70	65.8 ± 6.13	Qigong	Intervention	①②⑤⑥⑦	12 weeks, 5 times/week, 60 min/session
Yin et al. (2025) (24)	China	25/26	58.15 ± 7.09	58.8 ± 8.61	Qigong	Usual activity	①④	12 weeks, 5 times/week, 30 min/session

The study by Wang et al. (18) included different intervention durations for its groups; therefore, the groups were designated Wang Meihua 2023A and Wang Meihua 2023 B (the control group comprising 15 participants was the same). The study by Chen et al. (16) did not report an explicit intervention duration; hence, “not reported” was indicated. ① UPDRS-III; ② TUGT; ③ BBS; ④ Stride length; ⑤ Cadence; ⑥ Gait velocity; ⑦ PDQ39.

### 3.2 Risk of bias

**Figure 2:**
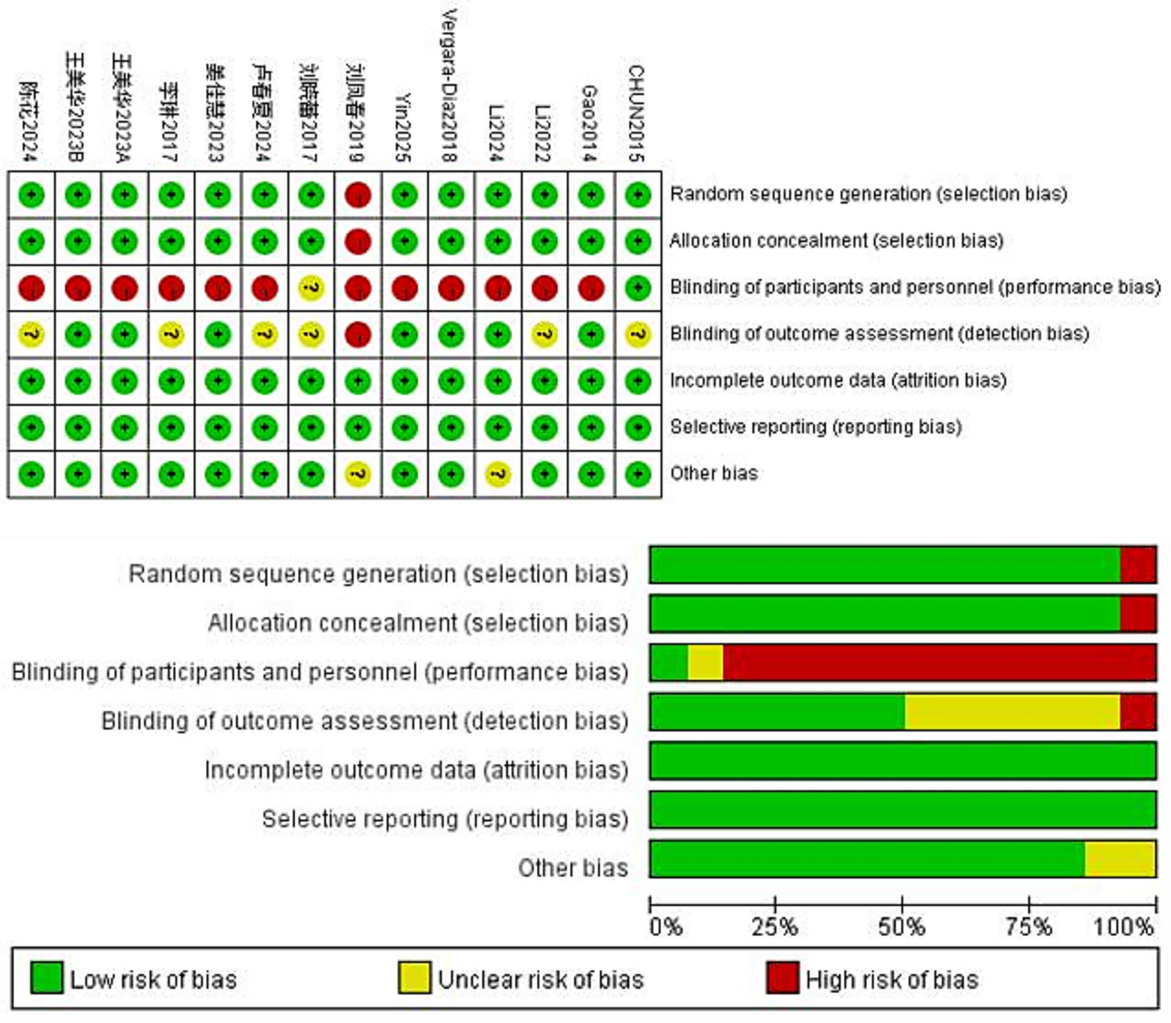
Risk of bias analysis for included studies.

Using the Cochrane version 5.1 risk of bias tool, two researchers (LL and HXY) independently assessed the risk of bias in the 13 included studies. Overall, the quality assessment indicated a moderate risk of bias. Figure 2 illustrates this risk-of-bias distribution. Twelve studies described the randomisation procedure, whereas one allocated participants through experimental and control groups using non-concurrent convenience sampling. Owing to the nature of TCEs, the blinding of participants and personnel was difficult to implement, which is a common limitation of such studies. In this systematic review and meta-analysis, only 11 studies reported obtaining informed consent from participants. Consequently, most studies showed a high risk of bias in the performance and detection bias domains.

### 3.3 Motor function

**Figure 3:**
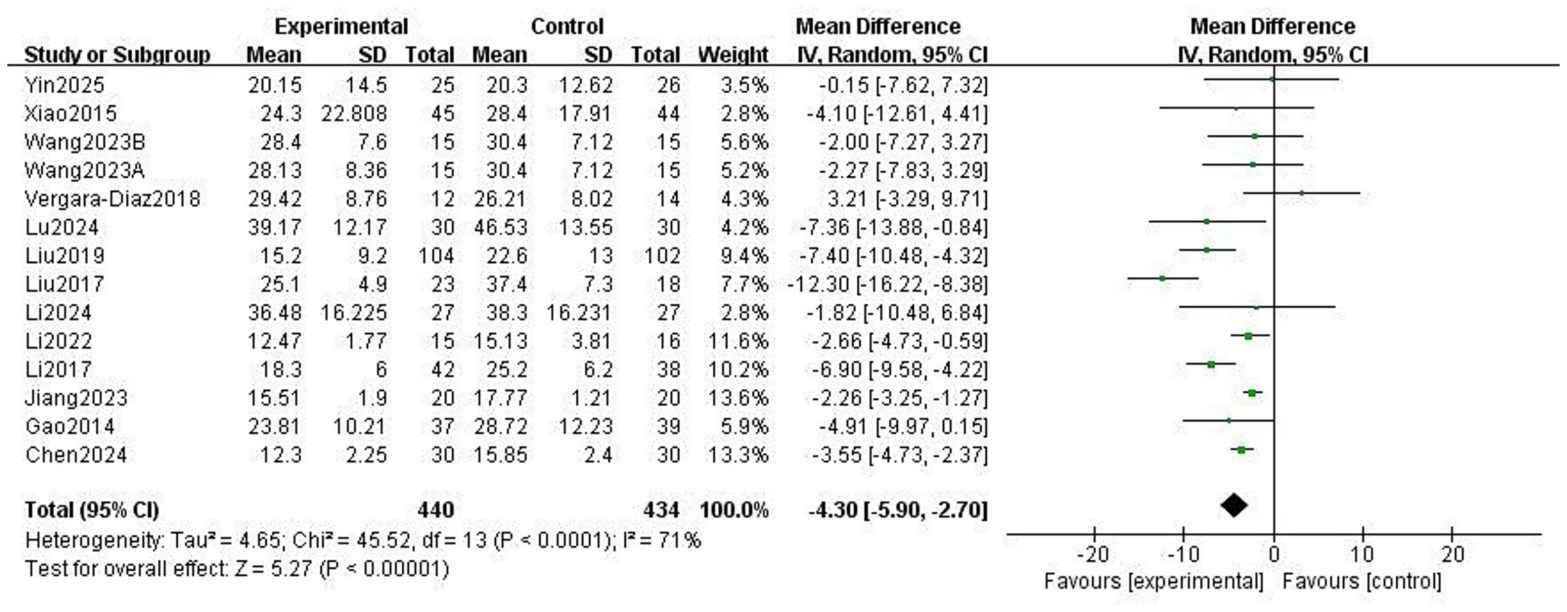
Effect of traditional Chinese exercises (TCEs) versus control on motor symptoms in patients with Parkinson’s disease (PD).

**Figure 4:**
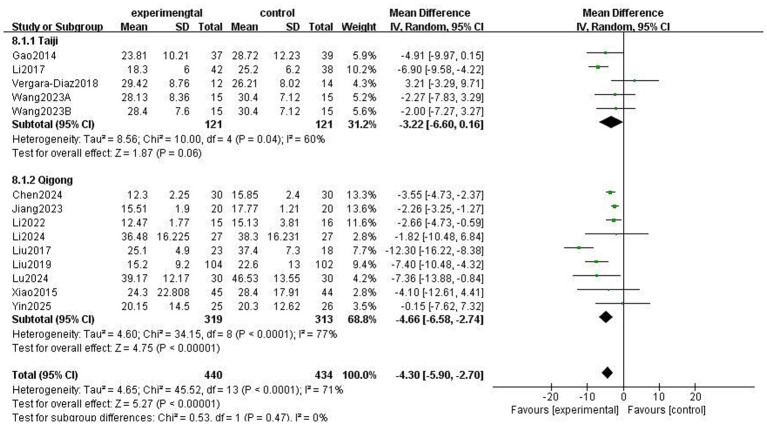
Effect of different exercise types on motor symptoms in patients with Parkinson’s disease (PD).

**Figure 5:**
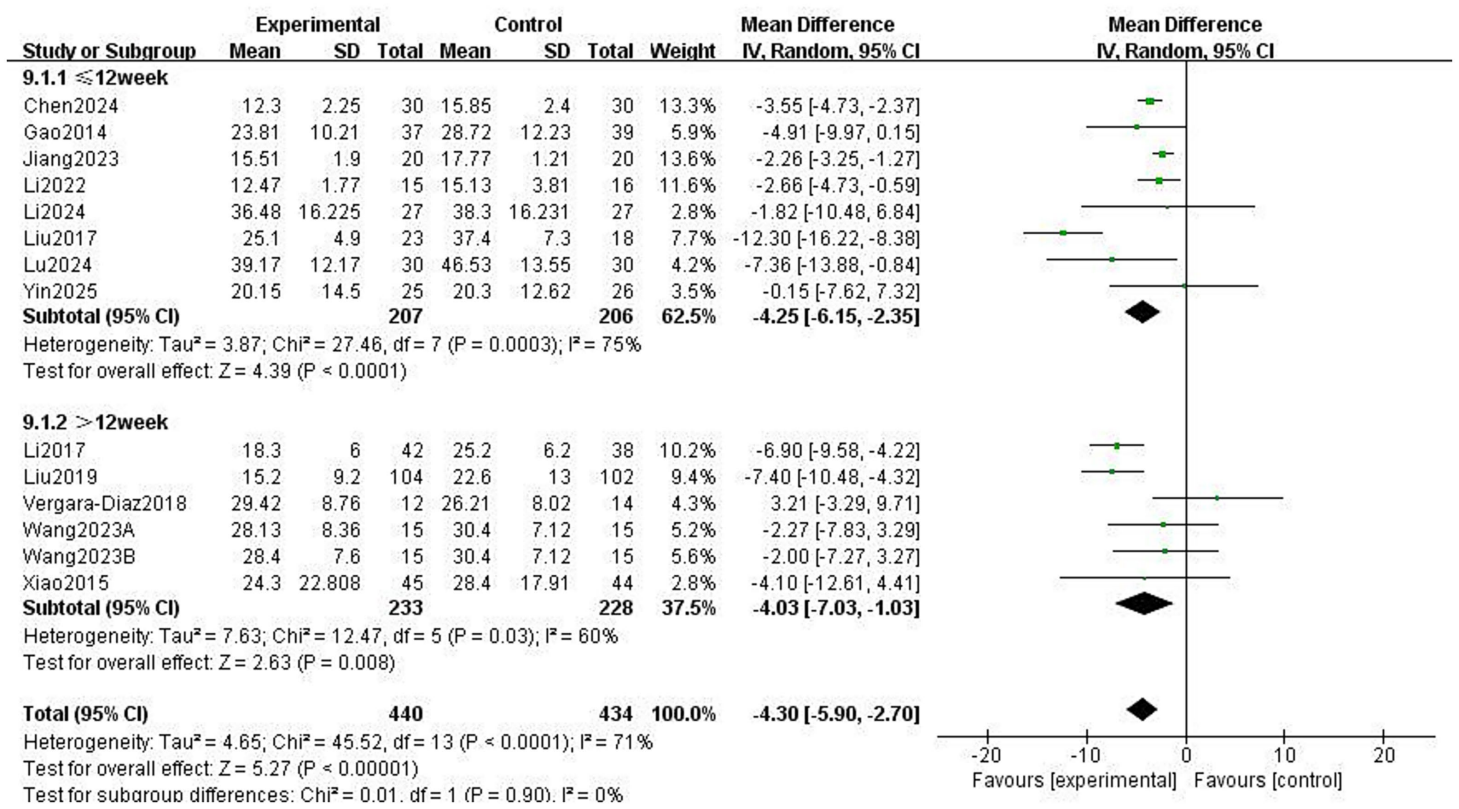
Effect of different exercise durations on motor symptoms in patients with Parkinson’s disease (PD).

**Figure 6:**
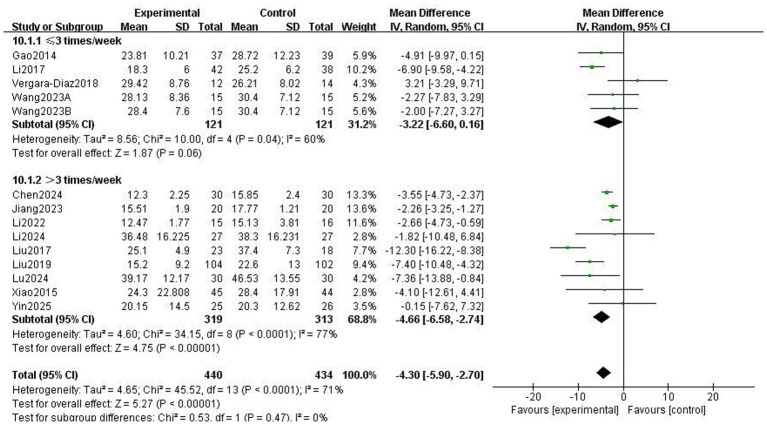
Effect of different exercise frequencies on motor symptoms in patients with Parkinson’s disease (PD).

**Figure 7:**
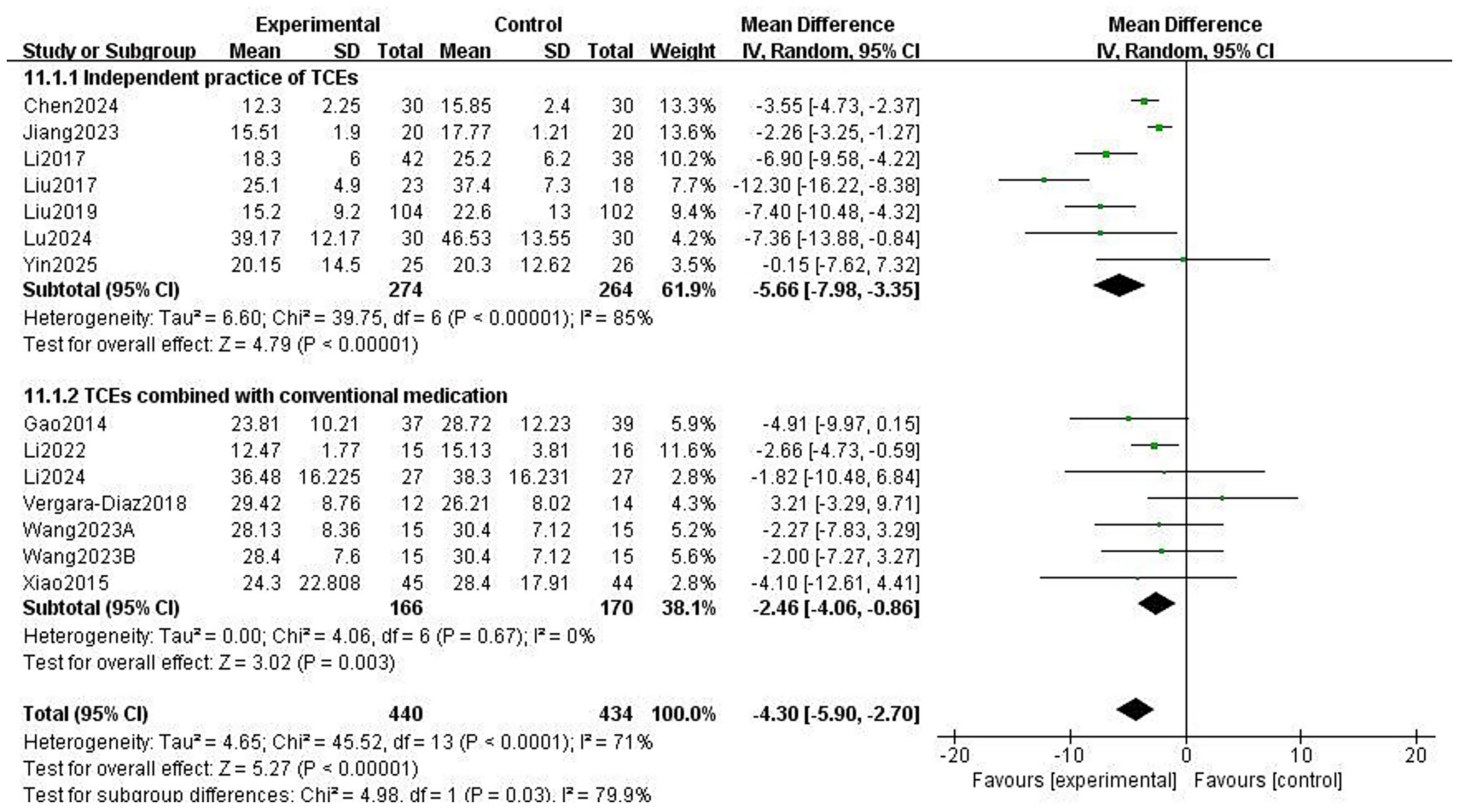
Effect of different intervention methods on motor ability in patients with Parkinson’s disease (PD).

The Unified Parkinson’s Disease Rating Scale Part III (UPDRS-III) is a standard clinical test for assessing the severity of motor complications in patients with PD, with a higher score indicating more severe motor complications (25). A total of 859 patients from 13 RCTs used UPDRS-III to evaluate motor function. The meta-analysis results showed that the UPDRS-III scores in the Traditional Chinese exercises (TCEs) intervention group were significantly lower than those in the control group (MD: −4.30, 95% CI: −5.90, −2.70, *p* < 0.00001), indicating a significant improvement in motor function. The heterogeneity among the studies was high (*I*^2^ = 71%). After sensitivity analysis, the heterogeneity significantly decreased to 53% when the study by Liu et al. (15) was excluded; however, the overall effect size remained unchanged (Figure 3). Subgroup analysis based on exercise type (Figure 4) showed that Tai Chi (MD: −3.22, 95% CI: −6.60 to 0.16, *p* = 0.06, *I*^2^ = 60%) and Qigong (MD: −4.66, 95% CI: −6.58 to −2.74, *p* < 0.00001, *I*^2^ = 77%) were analysed. The results indicated that Qigong significantly improved motor symptoms in patients with PD (*p* < 0.05). Subgroup analysis based on exercise duration (Figure 5) showed that ≤12 weeks (MD: −4.25, 95% CI: −6.15 to −2.35, *p* < 0.0001, *I*^2^ = 75%) and > 12 weeks (MD: −4.03, 95% CI: −7.03 to −1.03, *p* = 0.008, *I*^2^ = 60%) were analysed. The results indicated that an exercise duration of ≤12 weeks provided greater improvement in motor function for PD patients. Subgroup analysis based on exercise frequency (Figure 6) showed that ≤3 times (MD: −3.22, 95% CI: −6.60 to 0.16, *p* = 0.06, *I*^2^ = 60%) and >3 times per week (MD: −4.66, 95% CI: −6.58 to −2.74, *p* < 0.00001, *I*^2^ = 77%) were analysed. The results indicated that exercising > 3 times/week resulted in better motor improvements. Subgroup analysis based on the intervention method (Figure 7) showed that the independent practise of TCEs (MD: −5.66, 95% CI: −7.98, −3.35, *p* < 0.00001, *I*^2^ = 85%) and TCEs combined with conventional medication (MD: −2.46, 95% CI: −4.06, −0.86, *p* = 0.003, *I*^2^ = 0%) were analysed. The results indicated that independent practise of TCEs was more effective in improving mobility and reducing the risk of falls for PD patients with PD.

### 3.4 Balance function

**Figure 8:**
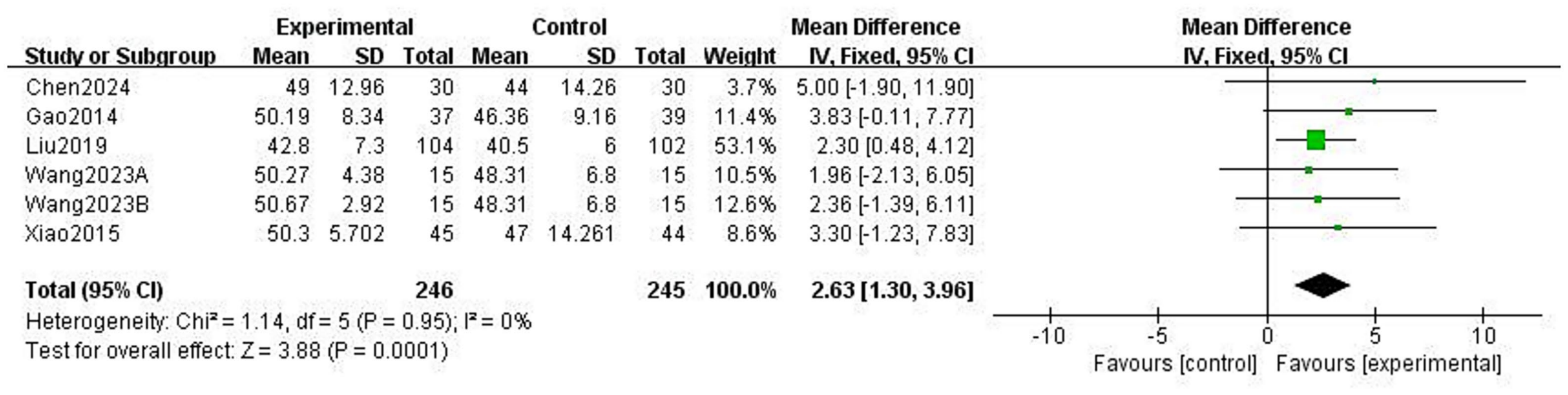
Effect of traditional Chinese exercises (TCEs) versus control on functional balance ability in patients with Parkinson’s disease (PD) (BBS).

**Figure 9:**
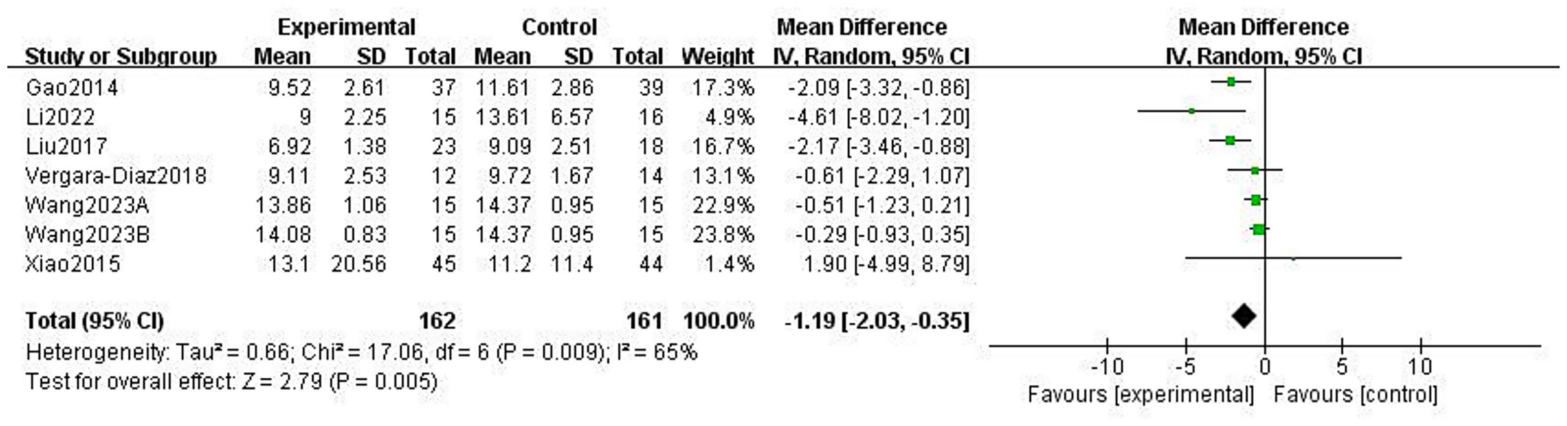
Effect of traditional Chinese exercises (TCEs) versus control on mobility balance and fall risk in patients with Parkinson’s disease (PD) (TUGT).

**Figure 10:**
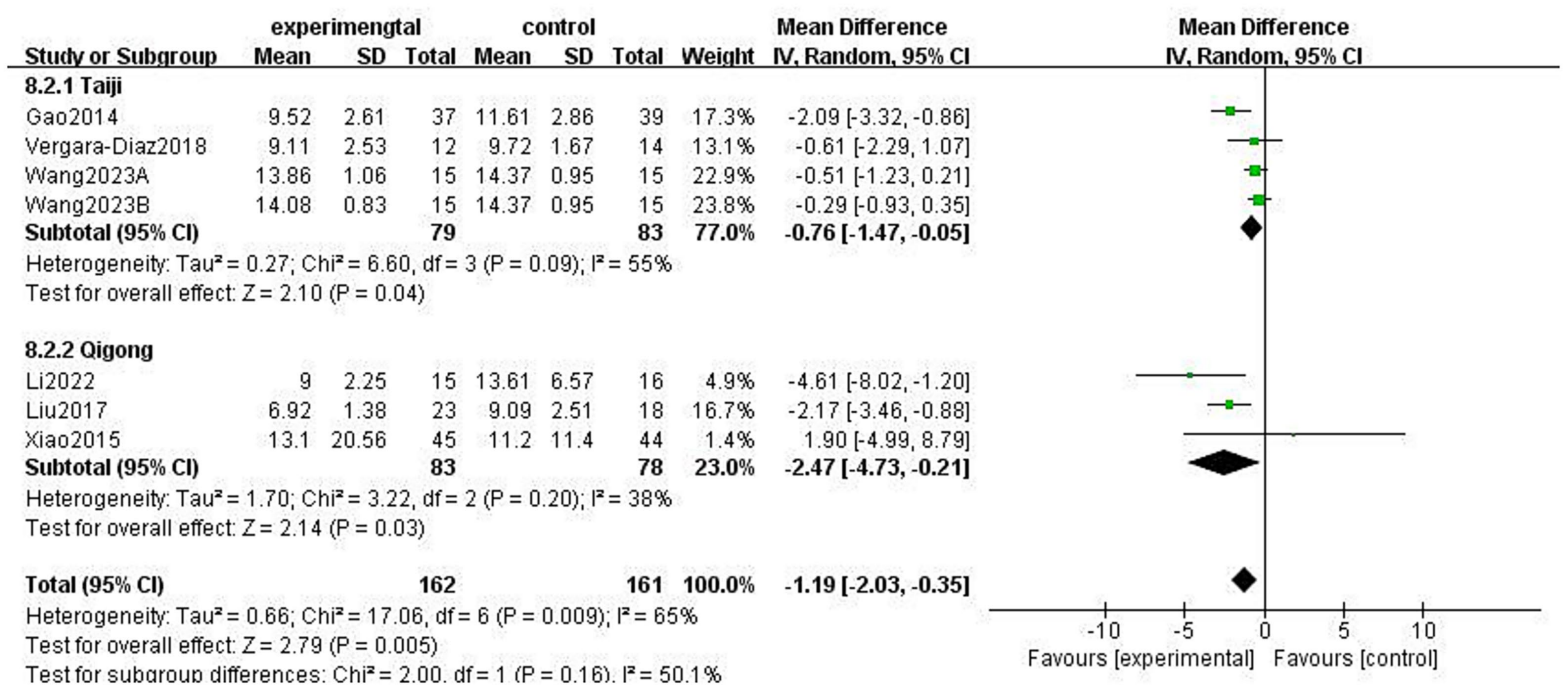
Effect of different exercise types on mobility balance and fall risk in patients with Parkinson’s disease (PD) (TUGT).

**Figure 11:**
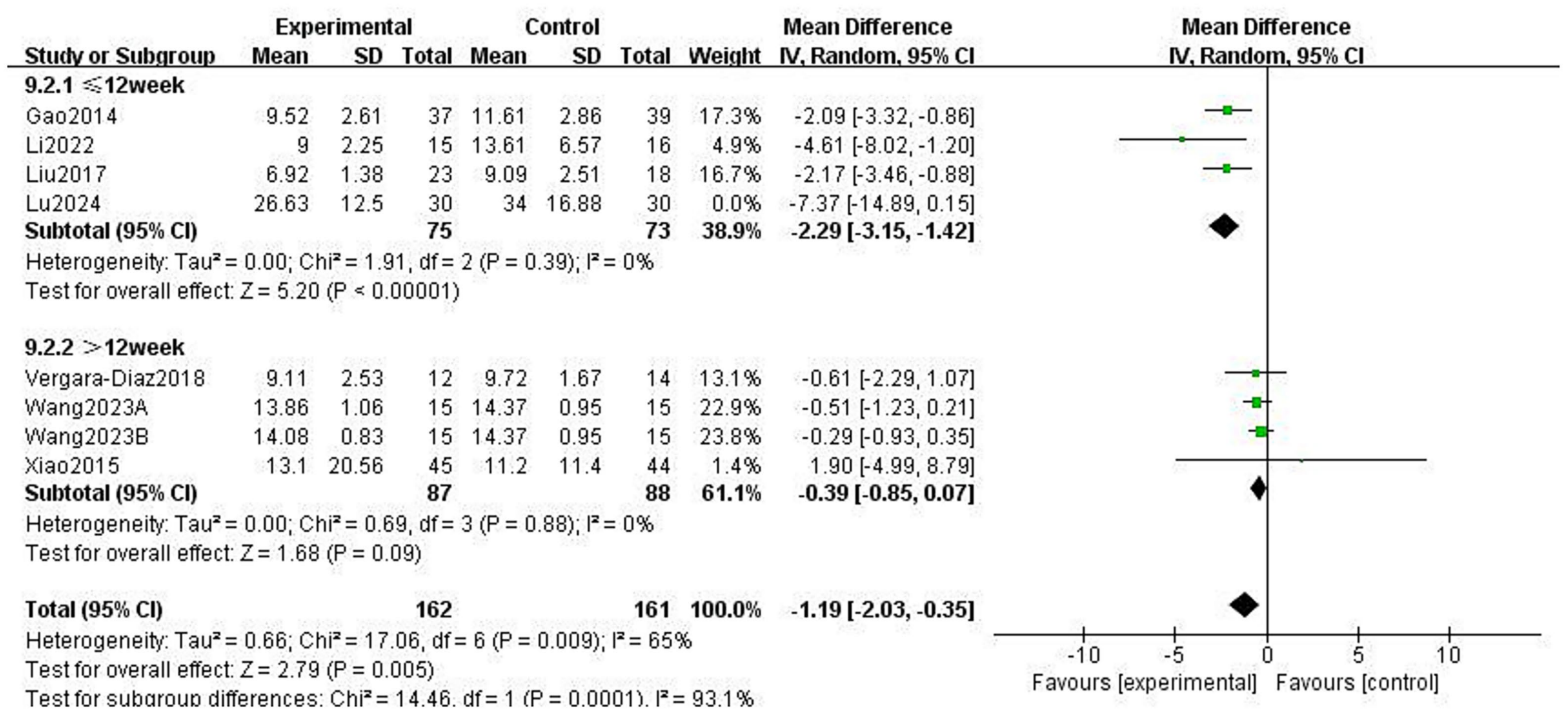
Effect of different exercise durations on mobility balance and fall risk in patients with Parkinson’s disease (PD) (TUGT).

**Figure 12:**
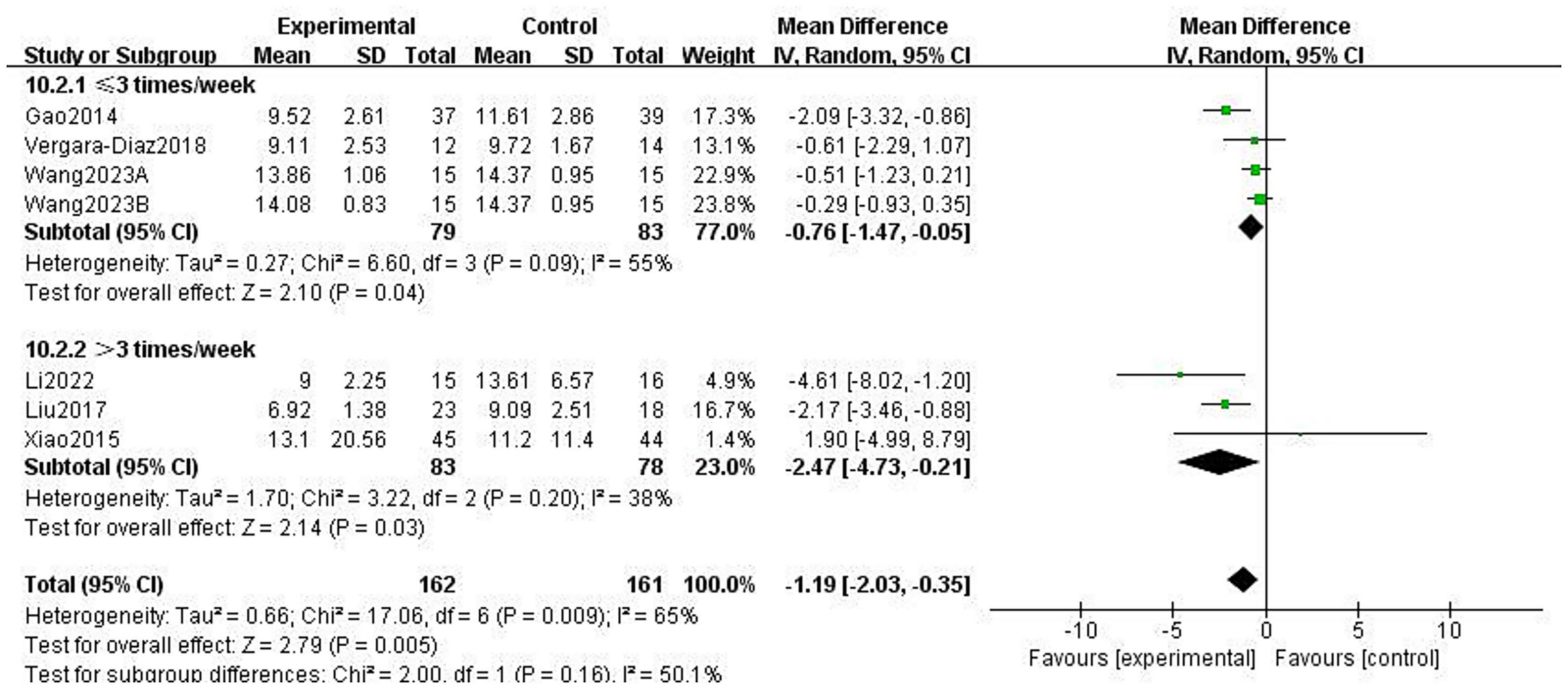
Effect of different exercise frequencies on mobility balance and fall risk in patients with Parkinson’s disease (PD) (TUGT).

This study used the Berg Balance scale (BBS) and the Timed Up and Go Test (TUGT) to evaluate balance function in patients with PD; both scales assess balance-related abilities but measure different aspects of functional stability. The BBS is used to assess functional balance and shows high validity and reliability in patients with PD, with a higher score indicating better balance. The Timed Up and Go Test (TUGT) is generally used to assess the risk of walking and falling in patients with PD, with a higher score indicating a higher risk. Meta-analysis data from five studies involving 476 patients utilised the BBS (Figure 8). Data using a fixed-effects model showed that TCEs significantly improved patients’ balance ability (MD: 2.63, 95% CI: 1.30 to 3.96, *p* = 0.0001, *I*^2^ = 0%). Six studies, involving 308 patients, used the TUGT scale. Data from a random-effects model (Figure 9) showed that the completion time for the intervention group was significantly reduced (MD: −1.19, 95% CI: −2.03, −0.35, *p* = 0.005, *I*^2^ = 65%), indicating that TCEs significantly improved mobility and reduced the risk of falls in patients with PD. Although this analysis had high heterogeneity (*I*^2^ = 65%), the sensitivity analysis revealed that heterogeneity decreased to 28% after excluding the studies by Wang et al. (18), and the conclusion remained significant. Subgroup analysis (Figures 10–12) showed that Tai Chi (MD: −0.76, 95% CI: −1.47 to −0.05, *p* = 0.04, *I*^2^ = 55%) and Qigong (MD: −2.47, 95% CI: −4.73 to −0.21, *p* = 0.03, *I*^2^ = 38%) were analysed. The results indicate that qigong provided better improvements in mobility and fall risk for patients with PD. For exercise duration, ≤12 weeks (MD: −2.29, 95% CI: −3.15 to −1.42, *p* < 0.00001, *I*^2^ = 0%) and >12 weeks (MD: −0.39, 95% CI: −0.85 to −0.07, *p* = 0.09, *I*^2^ = 0%) were analysed. The results showed that ≤12 weeks yielded a significant and stable effect on mobility and fall risk in PD patients. For exercise frequency, ≤3 times (MD: −0.76, 95% CI: −1.47 to −0.05, *p* = 0.04, *I*^2^ = 55%) and >3 times per week (MD: −2.47, 95% CI: −4.73 to −0.21, *p* = 0.03, *I*^2^ = 38%) were analysed. The results indicated that exercising >3 times per week was more effective in improving mobility and fall risk of patients with PD.

### 3.5 Gait function

**Figure 13:**
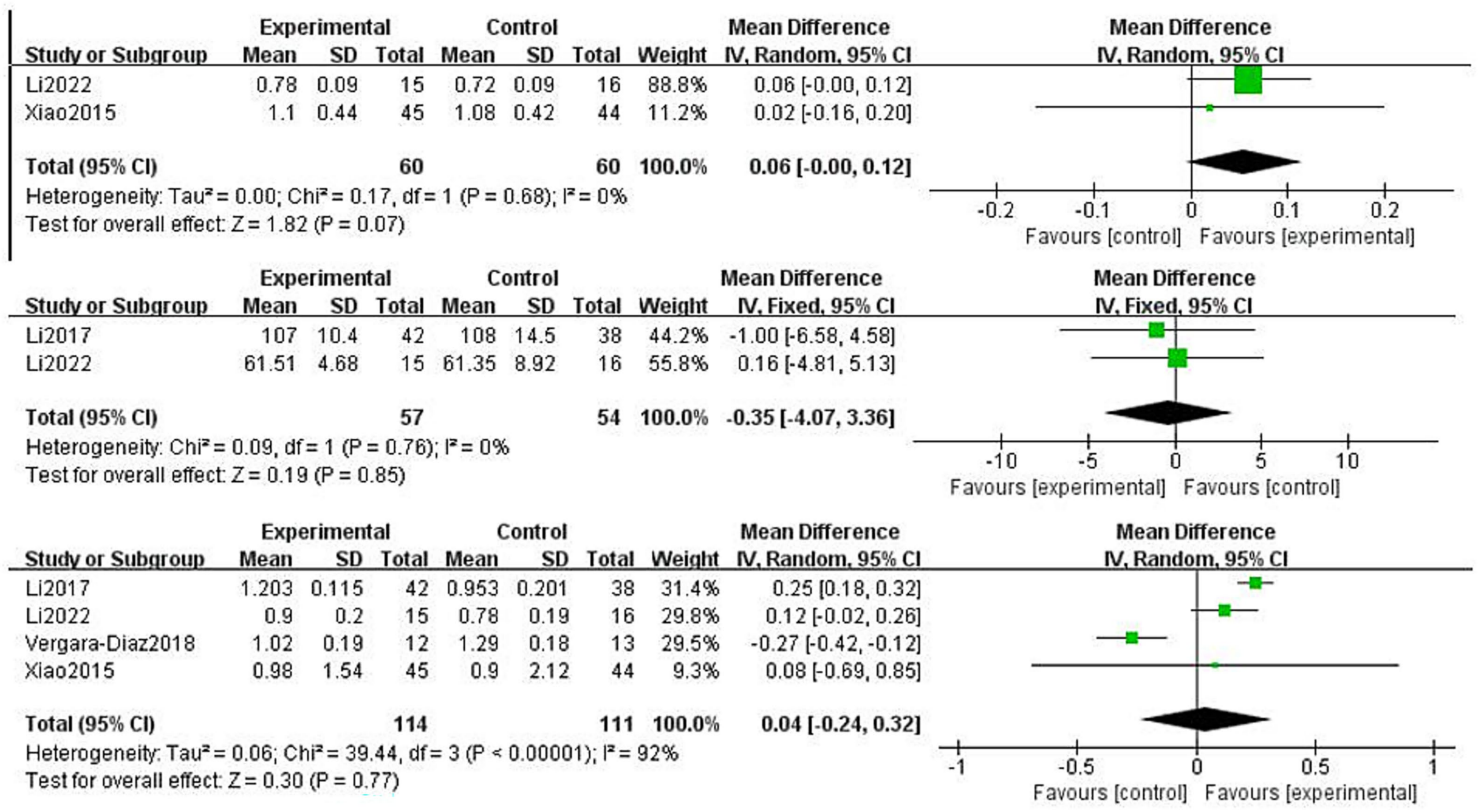
Effect of Traditional Chinese exercises (TCEs) versus control on gait ability in patients with Parkinson’s disease (PD) (Stride length, Cadence, Gait velocity).

Patients with PD experience difficulties in static-to-dynamic transitions, particularly during gait initiation, turning, and termination. In the four included studies, stride length, cadence, and gait velocity were selected as outcome measures to evaluate lower-limb gait function (Figure 13). Two studies on stride length involving 120 patients used a random-effects model, which showed no statistical significance (MD: 0.06, 95% CI: −0.00 to 0.12, *p* = 0.07, *I*^2^ = 0%). Two studies on cadence involving 111 patients used a fixed-effects model, which also showed no statistical significance (MD: −0.35, 95% CI: −4.07 to 3.36, *p* = 0.85, *I*^2^ = 0%). Four studies on gait velocity involving 225 patients used a random-effects model, which showed no significant differences (MD: 0.04, 95% CI: −0.24 to 0.32, *p* = 0.77, *I*^2^ = 92%). Sensitivity analysis showed that after excluding the study by Vergara-Diaz et al. (22), heterogeneity decreased to 29%, and the conclusion became statistically significant (*p* < 0.05). This change may be attributable to the small sample size and longer exercise duration of that study.

### 3.6 Quality of life

**Figure 14:**
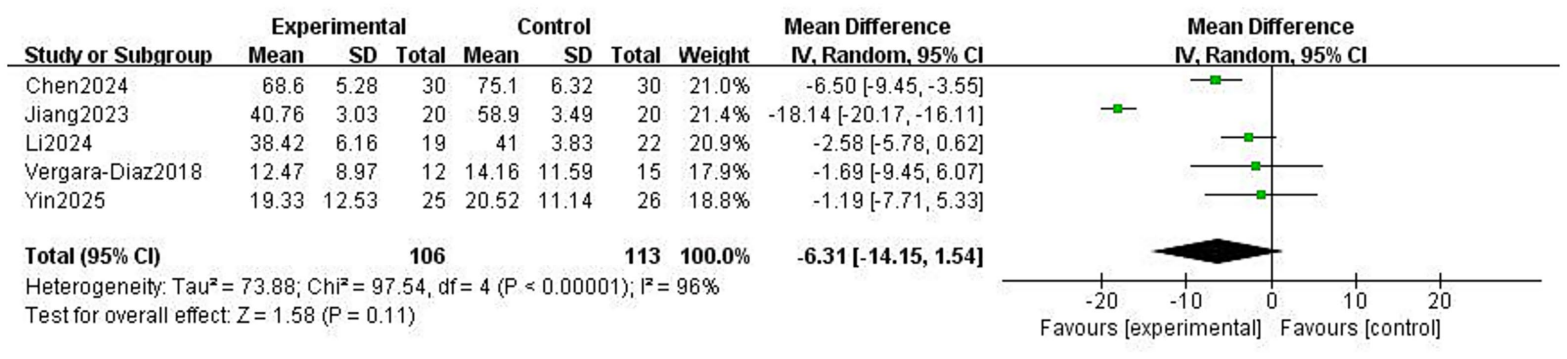
Effect of traditional Chinese exercises (TCEs) versus control on quality of life in patients with Parkinson’s disease (PD).

Quality of life is a crucial component reflecting a patient’s health status, which is also defined as “the impact of a disease or its adverse effects on a patient’s life based on personal perception and self-evaluation,” where a lower score represents a better quality of life for patients with PD (26). Five studies involving 232 patients used the PDQ-39 to assess the quality of life. As shown in Figure 14, the meta-analysis results indicated that TCEs did not result in a statistically significant improvement in quality of life (MD: −6.31, 95% CI: −14.15 to 1.54, *p* = 0.11, *I*^2^ = 96%). This suggests that TCEs do not significantly improve the quality of life in patients with PD. Sensitivity analysis was performed, and results showed that after excluding the Jiang 2023 study, heterogeneity decreased to 34% and became statistically significant (*p* < 0.05), suggesting that this study was the main source of heterogeneity. The reason for this was explored as the “personalised adaptation” innovation in the study methodology—adjusting intervention intensity by assessing fatigue symptoms before and after the intervention and linking fatigue improvement to enhanced quality of life—a design not utilised by the other studies.

### 3.7 Publication bias

**Figure 15:**
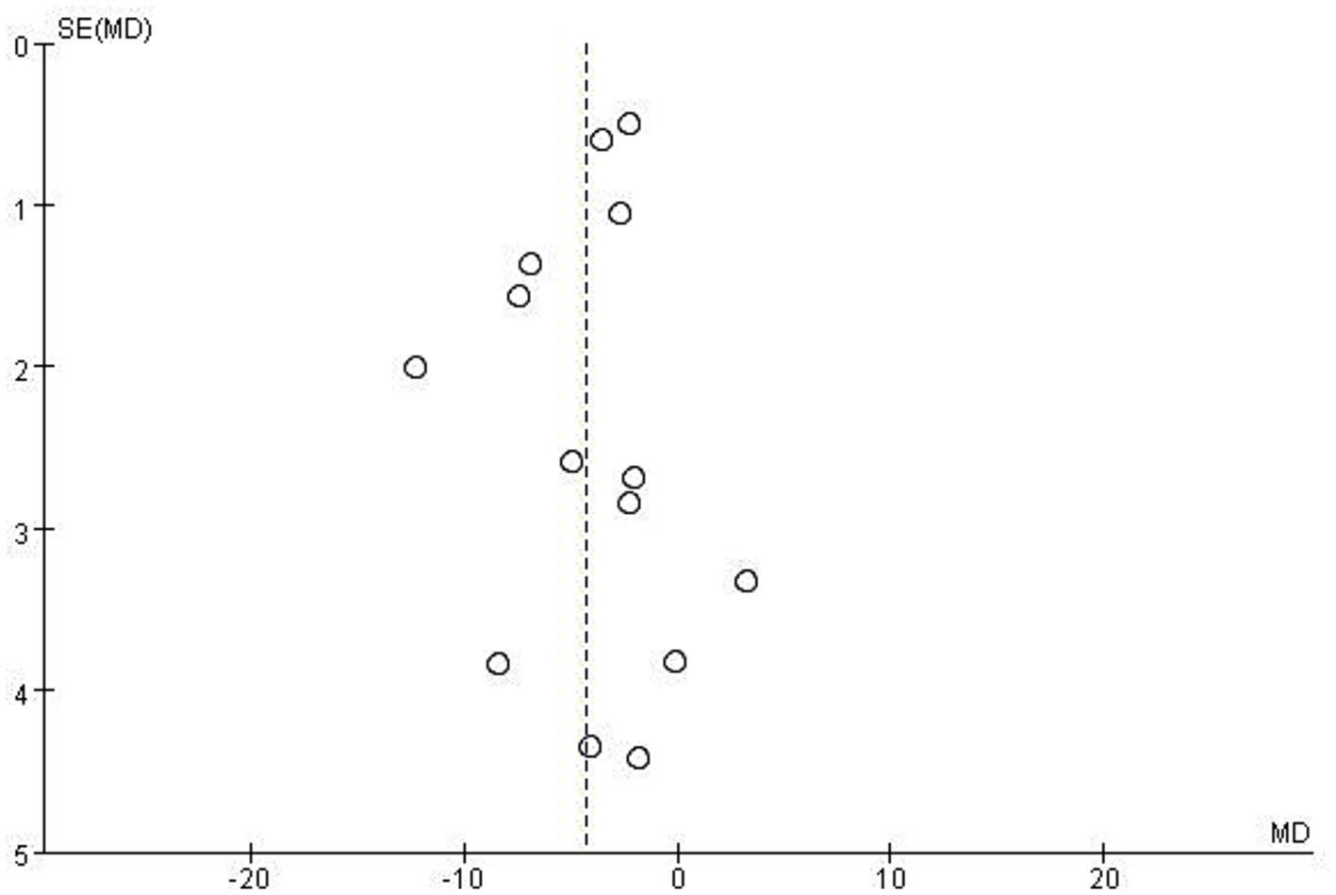
Funnel plot comparing the traditional Chinese exercises (TCEs) group and the control group.

A funnel plot test was conducted for the UPDRS-III, which included 10 studies (*n* = 13). As shown in Figure 15, the distribution of the included studies appeared asymmetrical, suggesting a potential publication bias in the RCTs for UPDRS-III scores. This finding indicates that studies with positive results may have been more likely to be published, potentially overestimating the overall effect size. Consequently, the overall methodological quality of the studies included in this review was considered to be low.

## 4 Discussion

This systematic review and meta-analysis included 13 randomised controlled trials (RCTs) involving 859 patients with PD to evaluate the effects of TCEs on motor function, balance, gait, and quality of life. The results indicated that TCEs significantly improved motor and balance functions in patients with PD but did not have a significant effect on gait function or quality of life. This conclusion confirms the findings of the latest relevant systematic review by Yuan et al. (27), which reported that traditional Chinese exercises improve motor and balance functions in patients with PD, and extends their work by assessing the efficacy on gait function and quality of life. The positive findings of this systematic review are primarily applicable to patients with mild-to-moderate PD diagnosed with Hoehn and Yahr stages 1–3, all of whom are capable of independently performing Tai Chi or Qigong exercises. The control group primarily received the usual care or no intervention. The included studies did not report additional benefits of combining TCEs with medication or rehabilitation robots on motor function in patients with PD. Subgroup analysis results suggest that this study recommends that early- and moderate-stage PD patients practise Qigong for a duration of 12 weeks, with a frequency of at least three times per week, and that independent practise of TCEs should be used as the optimal initial prescription.

### 4.1 Improvements in motor function

The meta-analysis results of this study indicate that TCEs significantly improves motor function in patients with PD, consistent with the findings of multiple prior studies (8, 28–30). The intervention group showed a statistically significant decrease in UPDRS-III scores compared to the control group (MD: −4.30, 95% CI: −5.90 to −2.70, *p* < 0.00001, *I*^2^ = 71%). This reduction was not only statistically significant (*p* < 0.05) but also implied a clinically meaningful alleviation of the patients’ motor symptoms. Motor dysfunction in patients with PD primarily stems from neurodegenerative changes in the central nervous system (31), leading to uncoordinated contraction of the agonist and antagonist muscles, which causes muscle tension and affects motor function (32). In response, TCEs, as a mind–body non-pharmacological therapy, may promote neuroplasticity or influence specific biomarkers (such as HIP2 mRNA) to regulate core neurodegenerative changes in PD through slow, controlled limb movements coordinated with breathing and intention (33). However, the subgroup analysis suggested the complexity of the intervention strategy: we found that the best effects were achieved with Qigong practise lasting ≤12 weeks and high-frequency training. We speculate that this duration dependence may be related to neurotransmitter regulation. Research has shown that continuous aerobic exercise for 12 weeks or less can increase striatal D2 receptor density; however, if extended to 24 weeks, it may lead to receptor desensitisation (34). This finding differs from the views of some studies advocating the effectiveness of long-term training (35, 36). Furthermore, in the comparison of exercise types, the improvement effect of Tai Chi did not reach statistical significance. This may reflect differences in the establishment of early motor coordination between different exercise types, as beginners of Tai Chi may find it difficult to accurately grasp complex key movements and the essence of martial arts within a limited exercise period. This does not imply that Tai Chi is ineffective as an exercise modality. In contrast, Qigong is based on the key principles of “regulating the body, regulating the breath, and regulating the mind,” achieving harmony of Qi and blood by standardising posture, controlling breathing, and calming the mind, without complex martial arts requirements, thus demonstrating a better improvement effect on motor function in this study. In summary, the findings of this study not only confirm the clinical efficacy of TCEs, but also emphasise the precise impact of exercise type, duration, frequency, and intervention method, providing strong evidence-based support for TCEs.

### 4.2 Enhancement of balance function

This study found that TCEs significantly improved balance and mobility in patients with PD, which is consistent with the conclusions of Dong et al. (37). The meta-analysis results showed a significant increase in BBS scores in the intervention group (MD: 2.96, 95% CI: 2.23 to 3.69, *p* < 0.00001, *I*^2^ = 0%), confirming the robust practical clinical value of TCEs for static balance. A significant reduction in completion time on the TUGT was observed (MD: −1.21, 95% CI: −2.05 to −0.38, *p* = 0.004, *I*^2^ = 65%), indicating that the dynamic mobility and fall risk of PD patients were significantly improved. These results collectively confirm the positive role of TCEs in enhancing balance function in patients. This effect may stem from dual mechanisms: on the one hand, the exercises enhance proprioception and centre of gravity control through slow, multidirectional movements and focused attention; on the other hand, they may exert a neuroprotective effect by reducing inflammation and oxidative stress within the nervous system (38, 39). Furthermore, the TUGT analysis showed high heterogeneity (*I*^2^ = 65%). After conducting a sensitivity analysis, it was found that heterogeneity was potentially attributable to the small sample size in the study by Wang et al. (18). Subgroup analysis based on exercise type showed that Qigong provided a slightly better improvement in performance on the TUGT than Tai Chi. Additionally, for balance and mobility, exercise durations > 12 weeks did not show additional significant gains, which is consistent with the research of Yu et al. (27), who stated that Tai Chi did not significantly alter the dynamic mobility and fall risk of PD patients. Therefore, future research should increase the number of large-sample randomised controlled trials focusing on the independent practise of qigong within 12 weeks and with a frequency of greater than 3 times per week to provide suitable exercise prescriptions for balance function in patients with mild-to-moderate PD.

### 4.3 Controversy surrounding gait function

Despite the significant improvements in motor and balance functions, the meta-analysis results of this study failed to confirm that TCEs significantly improved gait function (including stride length, cadence, and gait velocity) in patients with PD. Based on the meta-analysis results of this study, improvements for stride length (*p* = 0.07), cadence (*p* = 0.85), and gait velocity (*p* = 0.77) were not statistically significant, a finding that warrants further discussion. However, heterogeneity in the gait velocity meta-analysis was high (*I*^2^ = 92%). After sensitivity analysis, excluding the Vergara-Diaz et al. (22) study, heterogeneity was significantly reduced to 29%, and the result became statistically significant (*p* < 0.05). This suggests that the potential improvement effect of TCEs on gait may have been masked by a few heterogeneous studies. Some studies have confirmed that Tai Chi can improve knee flexion angle, thereby increasing stride length and gait velocity (40). Other research, by mimicking the “Bear Play” and “Monkey Play” movements in Wuqinxi (Five-Animal Play), found an increase in gait velocity when performing a single task (41). Furthermore, some studies have shown the positive effects of Baduanjin (Eight-Section Brocade) training on gait velocity (42), and some studies have indicated that Qigong is superior to yoga and dancing in improving gait velocity (10). These findings are inconsistent with the conclusions of the present systematic review. Moreover, some studies have also pointed out that Tai Chi has no significant effect on cadence and that Tai Chi combined with medication did not show better effects on gait velocity and stride length than medication alone (43, 44). The reasons for these discrepancies may be the limited sample size included in this study and the lack of standardisation of the testing techniques used across different studies. Furthermore, gait problems in patients with PD, particularly festinating or freezing of gait, are among the core motor impairments that may require specialised and precise intervention. Future research should consider expanding the sample size, adopting standardised testing techniques (such as wearable sensors or three-dimensional motion capture systems), and exploring the combination of TCEs with specific gait training methods, such as rehabilitation robot-assisted training (45).

### 4.4 Limitations in quality of life

Quality of life is a complex and multidimensional concept influenced not only by motor symptoms but also by non-motor symptoms, psychological health, and social support. Our preliminary analysis showed that TCEs did not demonstrate a statistically significant improvement in the quality of life of patients with PD (*p* = 0.11). Notably, the heterogeneity in this analysis was extremely high (*I*^2^ = 96%). Sensitivity analysis suggested that after excluding the Jiang 2023 study, which had a core difference due to its “personalised adaptation” intervention method (e.g., adjusting intensity based on pre- and post-intervention fatigue symptoms), heterogeneity was significantly reduced (*I*^2^ = 34%), and the effect size became significant (*p* < 0.05). This indicates that TCEs may have a potentially beneficial effect on quality of life. Nevertheless, this study’s result contradicts the conclusion that long-term practise of Tai Chi or Qigong can enhance quality of life by improving movement and alleviating anxiety (8, 46). Furthermore, subgroup analysis found that the optimal time for motor symptom improvement is ≤12 weeks, whereas the optimal time range for improving quality of life is typically between 13 and 24 weeks (47). This contrast suggests that prolonged single-sport intervention may weaken its cumulative effect on complex non-motor symptoms and overall quality of life because it fails to keep pace with the progression of PD. Therefore, integration with conventional drug therapy is needed to maintain efficacy. Some studies also point out that music therapy is more advantageous in improving the quality of life of patients with PD, followed by dance therapy, because dance can rapidly activate the basal ganglia and promote dopamine secretion (48). Therefore, future research should attempt to combine TCEs with other interventions, such as music therapy, to explore their potential to improve the quality of life of patients with PD.

### 4.5 Study limitations

To our knowledge, this study is one of the most comprehensive and systematic meta-analyses on TCEs as an intervention for patients with PD in recent years. Our strengths include strict adherence to the Preferred Reporting Items for Systematic Reviews and Meta-Analyses (PRISMA) guidelines, searching multiple domestic and international databases, and conducting comprehensive meta-analyses of several key outcome measures, thus providing the latest reliable evidence-based support for clinical decision-making. However, this study has several limitations. First, only one of the included studies used a double-blind procedure. This may have resulted in a high risk of performance and detection bias in most studies, weakening the stability and credibility of the results. Furthermore, the funnel plot presented asymmetrical characteristics, suggesting a potential publication bias that may lead to an overestimation of the overall effect size (MD: −4.30). Second, inconsistencies existed in the experimental design of the included studies. Future research should standardise the experimental design and unify the outcome measures. Third, our analysis was based only on published RCTs, and the sample sizes were generally small, indicating that some results should be interpreted with caution. Finally, although we performed sensitivity and subgroup analyses because of the high heterogeneity between studies, unavoidable differences between studies, such as exercise frequency, patient disease severity, and adherence, may have influenced the results.

## 5 Conclusion

The findings of this study indicate that TCEs, as a safe and effective non-pharmacological therapy, can substantially improve motor and balance functions in patients with PD, serving as a valuable adjunct to conventional therapies. Based on these findings, this study recommends that early- and moderate-stage patients with PD practise Qigong for a duration of 12 weeks, with a frequency of no less than three times per week, and that independent practise of TCEs should be used as the optimal initial prescription. However, since this analysis suggested that the improvement effect on gait function and quality of life was limited, and given the presence of some risk of bias and high heterogeneity, clinical application should be approached with caution. Future research should focus on developing standardised exercise prescriptions and exploring the potential for improvement in gait and long-term quality of life through large-sample multicentre randomised controlled trials, particularly by extending the intervention period and unifying the research design to further promote the clinical translation and widespread application of TCEs.

## References

[1] Global, regional, and national burden of Parkinson’s disease, 1990-2016: a systematic analysis for the global burden of disease study 2016. Lancet Neurol. (2018) 17:939–53. doi: 10.1016/S1474-4422(18)30295-3, 30287051 PMC6191528

[2] Bhalsing KS Abbas MM Tan LCS. Role of physical activity in Parkinson’s disease. Ann Indian Acad Neurol. (2018) 21:242–9. doi: 10.4103/aian.AIAN_169_18, 30532351 PMC6238554

[3] Gammon K. Neurodegenerative disease: brain windfall. Nature. (2014) 515:299–300. doi: 10.1038/nj7526-299a, 25396246

[4] Gao S Kaudimba KK Cai J Tong Y Tian Q Liu P . A mobile phone app-based tai chi training in Parkinson’s disease: protocol for a randomized controlled study. Front Neurol. (2020) 11:615861. doi: 10.3389/fneur.2020.615861 33519695 PMC7838616

[5] Olanow CW Stocchi F Lang AE. Current and emerging therapies for motor complications in Parkinson's disease. Mov Disord. (2013) 28:221–35. doi: 10.1016/S1474-4422(06)70521-X

[6] Tinazzi M Gandolfi M Artusi CA Bannister K Rukavina K Brefel-Courbon C . Advances in diagnosis, classification, and management of pain in Parkinson’s disease. Lancet Neurol. (2025) 24:331–47. doi: 10.1016/S1474-4422(25)00033-X, 40120617

[7] Yu Y. A network meta-analysis of four kinds of traditional Chinese fitness exercises for osteoporosis in middle-aged and elderly people. China Sport Sci Technol. (2020) 56:37–44. doi: 10.16470/j.csst.2020115

[8] Chen SH Zhang YJ Wang YT Chen S Zhang Y Liu X . The effect of qigong-based therapy on patients with Parkinson’s disease: a systematic review and meta-analysis. Clin Rehabil. (2020) 34:1436–48. doi: 10.1177/0269215520946695, 32727214

[9] Lan C Lai J Chen S Wong M. Tai chi Chuan to improve muscular strength and endurance in elderly individuals: a pilot study. Arch Phys Med Rehabil. (2000) 81:604–7. doi: 10.1016/S0003-9993(00)90042-X, 10807099

[10] Mustafaoglu R Ahmed I Pang MYC. Which type of mind–body exercise is most effective in improving functional performance and quality of life in patients with Parkinson’s disease? A systematic review with network meta-analysis. Acta Neurol Belg. (2022) 122:1433–46. doi: 10.1007/s13760-022-02070-4, 36056269

[11] Yang Y Qiu WQ Hao YL Lv ZY Jiao SJ Teng JF. The efficacy of traditional Chinese medical exercise for Parkinson’s disease: a systematic review and meta-analysis. PLoS One. (2015) 10:e0122469. doi: 10.1371/journal.pone.0122469, 25830664 PMC4382160

[12] Jiang J Bi H. The effect of health qigong Yijinjing on fatigue and quality of life in patients with mild to moderate Parkinson’s disease. Nurs Res. (2023) 37:4452–7. doi: 10.12102/j.issn.1009-6493.2023.24.017

[13] Lu C Zheng Y Mo X . Effect of Baduanjin combined with G-EO robot on motor function in 30 patients with Parkinson’s disease. Hunan J Tradit Chin Med. (2024) 40:18–21. doi: 10.16808/j.cnki.issn1003-7705.2024.04.005

[14] Liu F Chang H Meng Q . Specialized management of nursing quality based on motor symptoms of Parkinson's disease patients. Nurs Res. (2019) 33:2832–5. doi: 10.12102/j.issn.1009-6493.2019.16.022

[15] Liu X Wan Z Shang M . Efficacy observation of health qigong exercise on Parkinson's disease patients. Chin J Neuroimmunol Neurol. (2017) 24:34–7. doi: 10.3969/j.issn.1006-2963.2017.01-009

[16] Chen H Qin Z. Analysis of the effect of Jiyiqingxintan combined with Wuqinxi exercise on Parkinson's disease. China Health Standard Management. (2024) 15:128–31. doi: 10.3969/j.issn.1674-9316.2024.22.031

[17] Li L Yu L Wang D . The effect of tai chi exercise on gait and postural control in patients with mild to moderate Parkinson's disease. Zhejiang Med J. (2017) 39:535–8. doi: 10.12056/j.issn.1006-2785.2017.39.7.2016-1978

[18] Wang M Gan M Wu H . Effects of different training loads of tai chi on the rehabilitation of patients with early and middle stage Parkinson's disease. Prog Biochem Biophys. (2023) 50:2487–95. doi: 10.16476/j.pibb.2023.0297

[19] Gao Q Leung A Yang YH Yang Y Wei Q Guan M . Effects of tai chi on balance and fall prevention in Parkinson’s disease: a randomized controlled trial. Clin Rehabil. (2014) 28:748–53. doi: 10.1177/0269215514521044, 24519923

[20] Xiao CM Zhuang YC. Effect of health Baduanjin qigong for mild to moderate Parkinson’s disease. Geriatr Gerontol Int. (2016) 16:911–9. doi: 10.1111/ggi.12571, 26310941

[21] Li KF Li J Xia AL Wang XW Wang AL Shi Y . The effects of Baduanjin on fine motor skills in mild and moderate Parkinson’s disease: a randomized controlled trial. Clin Parkinson Relat Disord. (2024) 11:100276. doi: 10.1016/j.prdoa.2024.100276, 39502276 PMC11535373

[22] Vergara-Diaz G Osypiuk K Hausdorff JM Bonato P Gow BJ Miranda JGV . Tai chi for reducing dual-task gait variability, a potential mediator of fall risk in Parkinson’s disease: a pilot randomized controlled trial. Glob Adv Health Med. (2018) 7:2164956118775385. doi: 10.1177/2164956118775385, 29796338 PMC5960860

[23] Li XY Lv CF Liu XL Qin X. Effects of health qigong exercise on lower limb motor function in Parkinson’s disease. Front Med. (2021) 8:809134. doi: 10.3389/fmed.2021.809134 PMC8892581 35252225

[24] Yin HM Cheng OM Zhang X Yin H Cheng O Quan F . Effects of Liuzijue qigong on respiratory function among patients with Parkinson’s disease: a randomized clinical trial. BMC Complement Med Ther. (2025) 25:63. doi: 10.1186/s12906-025-04773-6, 39979896 PMC11843974

[25] Hssayeni MD Jimenez-Shahed J Burack MA Ghoraani B. Ensemble deep model for continuous estimation of unified Parkinson’s disease rating scale III. Biomed Eng Online. (2021) 20:32. doi: 10.1186/s12938-021-00872-w, 33789666 PMC8010504

[26] Yang Y Li XY Gong L Zhu YL Hao YL. Tai chi for improvement of motor function, balance and gait in Parkinson’s disease: a systematic review and meta-analysis. PLoS One. (2014) 9:e102942. doi: 10.1371/journal.pone.0102942, 25047456 PMC4105461

[27] Yuan F Wang H. Traditional Chinese exercises for motor symptoms and mobility in patients with Parkinson’s disease: a systematic review and meta-analysis. Front Aging Neurosci. (2025) 17:1612913. doi: 10.3389/fnagi.2025.1612913, 40904344 PMC12401968

[28] Lai JH Cai YF Yang LY Lai J Cai Y Yang L . Effects of Baduanjin exercise on motor function, balance and gait in Parkinson’s disease: a systematic review and meta-analysis. BMJ Open. (2022) 12:e067280. doi: 10.1136/bmjopen-2022-067280, 36379643 PMC9668024

[29] Wu S Ding C Zheng J He J Li X Wang Y . Efficacy of traditional Chinese exercise in improving gait and balance in cases of Parkinson’s disease: a systematic review and meta-analysis. Front Aging Neurosci. (2022) 14:927315. doi: 10.3389/fnagi.2022.927315 35847669 PMC9285003

[30] Lee C Fan S Ma C Liu C Chen S Lee P . Traditional Chinese exercises for motor symptoms and mobility in patients with Parkinson’s disease: a systematic review and meta-analysis. Clin Rehabil. (2020) 34:1215–29. doi: 10.1177/0269215520935581 PMC12401968 40904344

[31] Hammarlund CS Andersson K Andersson M Nilsson MH Hagell P. The significance of walking from the perspective of people with Parkinson’s disease. J Parkinsons Dis. (2014) 4:657–63. doi: 10.3233/JPD-140399, 25147140

[32] Xumin J Jiaqi G Yuanyuan Q . The inheritance and innovation of guidance techniques can be seen from the comparative study of several modern common guidance techniques. Lishizhen Med Mater Med Res. (2022) 33:444–6. doi: 10.3969/j.issn.1008-8849.2022.02.046

[33] Li G Huang P Cui SS . Mechanisms of motor symptom improvement by long-term tai chi training in Parkinson’s disease patients. Transl Neurodegener. (2022) 11:6. doi: 10.1186/s40035-021-00281-9 35125106 PMC8819852

[34] Petzinger GM Fisher BE McEwen S Beeler JA Walsh JP Jakowec MW. Exercise-enhanced neuroplasticity targeting motor and cognitive circuitry in Parkinson’s disease. Lancet Neurol. (2013) 12:716–26. doi: 10.1016/S1474-4422(13)70123-6, 23769598 PMC3690528

[35] Song R Grabowska W Park M Osypiuk K Vergara-Diaz GP Bonato P . The impact of tai chi and Qigong mind-body exercises on motor and non-motor function and quality of life in Parkinson’s disease: a systematic review and meta-analysis. Parkinsonism Relat Disord. (2017) 41:3–13. doi: 10.1016/j.parkreldis.2017.05.019, 28602515 PMC5618798

[36] Yan J Yang Z Zhang J Wu W Jiang F Li G. Effect of tai chi on motor function in Parkinson’s disease: a systematic review and meta-analysis of randomized controlled trials. Parkinsonism Relat Disord. (2022) 94:1–10.34844021

[37] Dong S Wang Y Wei H Du S Li X Zhu J . Effects of Baduanjin Exercise on Rehabilitation of Patients With Mild to Moderate Parkinson’s Disease. Front. Aging Neurosci. (2021) 13:791724. doi: 10.3389/fnagi.2021.791724 PMC8811304 35126049

[38] Lou LJ Xiang CY Hu YL Lou L Xiang C Hu Y . Tai chi improves balance, mobility and gait function of the lower limbs in patients with Parkinson’s disease: a systematic review and meta-analysis. Eur J Med Res. (2025) 30:107. doi: 10.1186/s40001-024-02151-5, 39962570 PMC11831811

[39] Wayne PM Gow BJ Costa MD Peng CK Lipsitz LA Hausdorff JM . Complexity-based measures inform effects of tai chi training on standing postural control: cross-sectional and randomized trial studies. PLoS One. (2014) 9:e114731. doi: 10.1371/journal.pone.0114731, 25494333 PMC4262457

[40] Zhu YZ Zhang J Zeng YJ. Overview of tyrosine hydroxylase in Parkinson’s disease. CNS Neurol Disord Drug Targets. (2012) 11:350–8. doi: 10.2174/187152712800792901, 22483316

[41] Tuon T Valvassori SS Dal Pont GC Paganini CS Pozzi BG Luciano TF . Physical training prevents depressive symptoms and a decrease in brain-derived neurotrophic factor in Parkinson’s disease. Brain Res Bull. (2014) 108:106–12. doi: 10.1016/j.brainresbull.2014.09.006, 25264157

[42] Li FZ Harmer P Fitzgerald K Li F Eckstrom E Stock R . Tai chi and postural stability in patients with Parkinson’s disease. N Engl J Med. (2012) 366:511–9. doi: 10.1056/NEJMoa1107911, 22316445 PMC3285459

[43] Li ZL Wang T Shen MY Li Z Shen M Song T . Comparison of wuqinxi qigong with stretching on single- and dual-task gait, motor symptoms and quality of life in Parkinson’s disease: a preliminary randomized control study. Int J Environ Res Public Health. (2022) 19:8042. doi: 10.3390/ijerph19138042, 35805699 PMC9265753

[44] Amano S Nocera JR Vallabhajosula S Juncos JL Gregor RJ Waddell DE . The effect of tai chi exercise on gait initiation and gait performance in persons with Parkinson’s disease. Parkinsonism Relat Disord. (2013) 19:955–60. doi: 10.1016/j.parkreldis.2013.06.007, 23835431 PMC3825828

[45] Hackney ME Earhart GM. Tai chi improves balance and mobility in people with Parkinson disease. Gait Posture. (2008) 28:456–60. doi: 10.1016/j.gaitpost.2008.02.005, 18378456 PMC2552999

[46] Zhang L-X Zhang X-C. Gait planning and kinematic simulation for a lower limb gait rehabilitation robot. J Bionic Eng. (2009) 6:1–8. doi: 10.1016/S1672-6529(09)60002-3

[47] Aras B Seyyar GK Fidan O Colak E. The effect of tai chi on functional mobility, balance and falls in Parkinson’s disease: a systematic review and meta-analysis of systematic reviews. Explore. (2022) 18:402–10. doi: 10.1016/j.explore.2021.12.002, 34952799

[48] Peng WQ Zhao RH Huang ZT Peng W Zhao R Huang Z . Efficacy of oriental exercises for non-motor symptoms and quality of life in Parkinson’s disease: a systematic review and meta-analysis. Am J Chin Med. (2024) 52:2233–54. doi: 10.1142/S0192415X24500861, 39722602

